# Protective effects of *Lactobacillus rhamnosus* GG supernatant on metabolic associated fatty liver disease through intestinal barrier restoration and regulation of the regenerating gene 3γ

**DOI:** 10.3389/fmicb.2025.1580171

**Published:** 2025-09-02

**Authors:** Si-Pu Wang, Ling Ba, Xin-Rui Lv, Ya-Xin Qi, Xu Wang, Jie Zhang, Xin Xu

**Affiliations:** ^1^Department of Gastroenterology and Hepatology, Tianjin Medical University General Hospital, Tianjin, China; ^2^Tianjin Institute of Digestive Disease, Tianjin Medical University General Hospital, Tianjin, China

**Keywords:** antimicrobial peptide, intestinal barrier, *Lactobacillus rhamnosus* GG, MAFLD, Reg IIIγ

## Abstract

**Objective:**

The regenerating gene 3γ (Reg IIIγ) protein, a key antimicrobial peptide, is essential for maintaining intestinal barrier homeostasis and host defense. Its expression is impaired in metabolic-associated fatty liver disease (MAFLD), particularly under high-fat diet (HFD) conditions, contributing to barrier dysfunction. Given evidence that probiotic-derived components can modulate Reg IIIγ, this study aimed to evaluate the effects of *Lactobacillus rhamnosus* GG supernatant (LGGs) on Reg IIIγ expression, their impact on intestinal barrier function, and their therapeutic potential in mitigating MAFLD, while elucidating the underlying mechanisms involving the TLR2/MyD88/pSTAT3 signaling pathway.

**Methods:**

Six-week-old C57BL/6 J mice were randomly assigned to four groups: standard diet with phosphate-buffered saline (PBS), standard diet with LGGs, high-fat diet (HFD) with PBS, and HFD with LGG. The expression of intestinal Reg IIIγ, changes in intestinal microbiota, and intestinal permeability were analyzed using quantitative PCR (qPCR) and western blot techniques. *In vitro* experiments involved assessing HIP/PAP expression in Caco-2 cell lines following stimulation with LGG supernatants, using qPCR and western blot. Additionally, siRNA transfection of Caco-2 cells was used to examine the MyD88/pSTAT3 signaling pathway.

**Results:**

HFD impaired the intestinal barrier in mice. However, oral administration of LGG significantly enhanced the expression of Reg IIIγ in the intestinal mucosa compared to control groups. This intervention notably improved intestinal barrier function, modulated the composition of intestinal microbiota, and mitigated MAFLD. Furthermore, an inverse correlation was observed between intestinal permeability and Reg IIIγ expression. *In vitro*, stimulation of Caco-2 cells with LGG led to a significant upregulation of HIP/PAP protein expression, mediated through the MyD88/pSTAT3 signaling pathway.

**Conclusion:**

LGG supernatant enhances intestinal Reg IIIγ expression through the MyD88/pSTAT3 signaling pathway, thereby contributing to the protection of intestinal barrier function and alleviation of MAFLD.

## 1 Introduction

Metabolic-associated fatty liver disease (MAFLD), affecting ~25% of adults globally (Eslam et al., 2020), is strongly linked to metabolic syndrome and poses significant risks for cirrhosis and hepatocellular carcinoma (Chalasani et al., 2018; Paul and Davis, 2018). Its pathogenesis involves complex interactions, with gut dysbiosis and impaired intestinal barrier function playing pivotal roles (Naik et al., 2013; Harley et al., 2014; Qi et al., 2020; Dubinkina et al., 2017).

A hallmark of MAFLD is increased intestinal permeability (“leaky gut”), facilitating translocation of bacteria and microbial products (e.g., lipopolysaccharide, LPS) to the liver via the portal vein, this triggers aberrant hepatic immune activation, inflammation, and fibrosis (Safari and Gérard, 2019; Zhu et al., 2013; Vindigni et al., 2016; Mohammad and Thiemermann, 2021). High-fat diet (HFD), a major environmental risk factor, critically disrupts the intestinal barrier through inducing epithelial inflammation, oxidative stress, and dysbiosis (e.g., reduced Bacteroidetes, increased Firmicutes), collectively promoting metabolic endotoxemia and MAFLD progression (Gulhane et al., 2016; Sonnenburg et al., 2016; Agus et al., 2016).

One of the key factors affecting intestinal barrier integrity and defense function is regenerative islet-derived protein 3γ (Reg IIIγ), an antimicrobial peptide highly expressed in the gut (Terazono et al., 1988; Keim et al., 1991; Fraunhoffer et al., 2021). Reg3γ promotes mucosal repair by restoring tight junction proteins, reducing epithelial cell apoptosis, and directly targeting Gram-positive bacteria (Mukherjee et al., 2014; Zhao et al., 2013; Chen et al., 2019; Vaishnava et al., 2011). Critically, HFD is known to suppress intestinal Reg3γ expression, exacerbating barrier dysfunction (Shin et al., 2022). The expression of Reg3γ is associated with the regulation of the Toll-like receptor 2 (TLR2) signaling pathway. TLR2 activation by microbial ligands engages the adaptor MyD88, leading to phosphorylation of Signal Transducer and Activator of Transcription 3 (pSTAT3), which directly transactivates the Reg IIIγ gene (26866937). Consequently, the dysregulation of the TLR2/MyD88/pSTAT3 signaling pathway may be implicated in the barrier dysfunction observed in metabolic-associated fatty liver disease (MAFLD).

Probiotic interventions, particularly *Lactobacillus rhamnosus* GG (LGG), show promise in ameliorating MAFLD (32981107, 30251018). Notably, the beneficial effects of probiotics may not solely depend on viable bacteria but also involve bioactive metabolites secreted into the culture supernatant. Studies suggest that specific molecules derived from Lactobacillus can enhance Reg IIIγ expression (Frazier et al., 2022). However, whether LGG-s exerts its protective effects in MAFLD via modulating the TLR2/MyD88/pSTAT3 signaling pathway to upregulate Reg IIIγ remains incompletely elucidated.

Therefore, this study aimed to investigate the therapeutic potential of LGGs in HFD-induced MAFLD mice, with a specific focus on its role in activating the TLR2/MyD88/pSTAT3 pathway, enhancing Reg IIIγ expression, and subsequently restoring intestinal barrier integrity and mitigating liver pathology.

## 2 Materials and methods

### 2.1 LGG culture and LGGs preparation

The LGG strain (ATCC 53103, United States) was obtained from the China General Microbiological Culture Collection Center. It was cultured in De Man, Rogosa, and Sharpe (MRS) broth (Solaibao, China) at 37°C under microaerophilic conditions. The culture medium was then centrifuged at 8,000 rpm for 15 min at 4°C to isolate the supernatant. The LGGs was filtered through a 0.22 μm membrane filter to ensure sterility prior to use. Finally, store it in a freezer at −80°C for subsequent experiments.

### 2.2 Cell culture and siRNA knockdown experiment

The human colon cancer cell line, Caco-2, and the rat small intestinal epithelial cell line, IEC-6, were obtained from the Cell Bank of the Chinese Academy of Sciences (Shanghai, China) for *in vitro* studies. Caco-2 cells were cultured in Dulbecco’s Modified Eagle Medium (Procell) supplemented with 20% fetal bovine serum (Procell) and 1% penicillin–streptomycin (Solarbio), and maintained at 37°C in a 5% CO₂ atmosphere. Similarly, IEC-6 cells were cultured in DMEM supplemented with 20% fetal bovine serum, 1% penicillin–streptomycin, and 0.01 mg/mL insulin (Novo Nordisk), under the same environmental conditions. Transfection of Caco-2 cells with siRNA targeting MyD88 was conducted using Lipofectamine 2000 (Invitrogen).

### 2.3 Animal treatment

All animal experiments were conducted in accordance with the guidelines approved by the Animal Care and Use Committee of the General Hospital of Tianjin Medical University (IRB2022-DWFL-447). C57BL/6 J mice, aged 6–8 weeks and weighing 20–22 grams, were obtained from the HuafuKang Experimental Animal Centre (Beijing, China). Prior to the experiments, the mice were acclimatized for 1 week to minimize potential environmental variability. They were housed in a specific pathogen-free (SPF) facility under controlled conditions, including a temperature of 22°C, humidity levels of 50–60%, and a 12-h light/dark cycle, with unrestricted access to water and food.

The mice were randomly assigned to four groups: the standard diet with phosphate-buffered saline (PBS) group, the standard diet with LGG supernatant group, the HFD with PBS group, and the HFD with LGGs group. The HFD groups were fed XTHF60-1 chow (Jiangsu Synergy Pharmaceutical Biological Engineering, Jiangsu, China) for 12 weeks to induce obesity and establish a diet-induced obese mouse model. In contrast, the standard diet groups received irradiated, sterilized laboratory maintenance chow.

Following the induction of the model, the LGG groups were administered an oral gavage of 0.2 mL of LGG supernatant every other day for 8 weeks, while the PBS groups received an equivalent volume of PBS. At the end of the experimental period, fecal samples were collected from each mouse. The mice were anesthetized by intraperitoneal injection of tribromoethanol at a dose of 400 mg/kg. Following anesthesia, the mice were euthanized by cervical dislocation for tissue collection, and all harvested tissues were stored at −80°C.

### 2.4 Biochemical analysis

The biochemical parameters of mouse serum, including alanine aminotransferase (ALT), aspartate aminotransferase (AST), total cholesterol (TC), triglycerides (TG), and low-density lipoprotein cholesterol (LDL-C), were measured using a fully automated biochemical analyzer (BC240vet, Mindray, China). The concentration of lipopolysaccharides (LPS) in mouse serum was quantified with an LPS-specific ELISA kit for mouse serum (GILED, Wuhan, China). Serum fluorescein isothiocyanate (FITC) levels were assessed after the administration of FITC-Dextran (Meilunbio, China) via oral gavage.

### 2.5 Oil red O staining

Liver tissues were promptly frozen and sectioned using a cryostat. The tissue sections were then fixed by immersion in 4% paraformaldehyde (Solaibao, China) and stained with Oil Red O. Excess nonspecific staining was removed by washing the sections with ethanol. Hematoxylin was applied for nuclear counterstaining, followed by dehydration through a graded ethanol series. The sections were subsequently cleared in xylene and mounted with neutral gum. Lipid deposition in the liver tissue was then examined under a light microscope (Leica, Germany).

### 2.6 HE staining, IHC staining, and TUNEL staining

After euthanasia, ileal tissues were collected, and the distal sections were prepared as swiss rolls and fixed in 4% formaldehyde. Colon tissues were embedded in paraffin and sectioned into 4 μm thick slides. The slides were fully dewaxed using xylene and ethanol and then stained with the conventional hematoxylin and eosin (H&E) technique. Images were captured using a light microscope (Leica, Germany).

The deparaffinized tissue slides were incubated overnight at 4°C with a Ki-67 primary antibody. The following day, the slides were washed three times with PBS and incubated with a secondary antibody at 37°C for 30 min. Images of the slides were subsequently captured using a light microscope (Leica, Germany).

To assess apoptosis, tissue sections were processed with a TdT-mediated dUTP Nick-End Labeling (TUNEL) assay kit. The stained sections were observed under a fluorescence microscope to assess apoptosis.

### 2.7 Total RNA extraction, reverse transcription, and real time quantitative PCR

Total RNA from colon tissues was extracted using TRIzol reagent (Qiagen, United States) according to the protocol provided by the manufacturer. After this, cDNA was synthesized through reverse transcription using the ABScript HII Reverse Transcriptase (Abclonal, China), following the instructions provided by the manufacturer. Quantitative PCR was subsequently performed using the ABclonal 2X Universal SYBR Green Fast qPCR Mix (Abclonal, China).

The relative expression levels of target genes were calculated using the2ΔΔCt method, with glyceraldehyde-3-phosphate dehydrogenase (GAPDH) serving as the internal control. The oligonucleotide sequences of the forward and reverse primers for each target gene are provided in Table 1.

**Table 1:** Primer sequences used for quantitative real-time PCR.

Primers	Sequence	Primer sequence
Srebf1	Forward	5′-CGACTACATCCGCTTCTTGCAG-3′
	Reverse	5′-CCTCCATAGACACATCTGTGCC-3′
Fasn	Forward	5′-CACAGTGCTCAAAGGACATGCC-3′
	Reverse	5′-CACCAGGTGTAGTGCCTTCCTC-3′
Acc	Forward	5′-GACAGACTGATCGCAGAGAAAG-3′
	Reverse	5′-TGGAGAGCCCCACACACA-3′
Scd1	Forward	5′-CCTCTTCGGGATTTTCTACTACATG-3′
	Reverse	5′-GCCGTGCCTTGTAAGTTCTGT-3′
IL-22	Forward	5′-GCTTGAGGTGTCCAACTTCCAG-3′
	Reverse	5′-ACTCCTCGGAACAGTTTCTCCC-3′
IL-6	Forward	5′-TACCACTTCACAAGTCGGAGGC-3′
	Reverse	5′-CTGCAAGTGCATCATCGTTGTTC-3′
IL-1β	Forward	5′-TGGACCTTCCAGGATGAGGACA-3′
	Reverse	5′-GTTCATCTCGGAGCCTGTAGTG-3′
TNF-α	Forward	5′-GGTGCCTATGTCTCAGCCTCTT-3′
	Reverse	5′-GCCATAGAACTGATGAGAGGGAG-3′
Reg3γ	Forward	5′-CGTGCCTATGGCTCCTATTGCT-3′
	Reverse	5′-TTCAGCGCCACTGAGCACAGAC-3′
HIP/PAP	Forward	5′-TATGGCTCCCACTGCTATGCCT-3′
	Reverse	5′-TCTTCACCAGGGAGGACACGAA-3′

### 2.8 Western blot analysis

Proteins were extracted from ileal tissues and cells using RIPA lysis buffer (Solarbio, China), supplemented with protease and phosphatase inhibitors, and stored at −80°C. The extracted proteins were separated by 7.5–10% SDS-polyacrylamide gel electrophoresis and transferred onto polyvinylidene difluoride (PVDF) membranes (Merck Millipore, Germany). The membranes were blocked with 5% non-fat milk for 1 h at room temperature.

Following the blocking step, the membranes were incubated overnight at 4°C with primary antibodies, as detailed in Table 2. The next day, the membranes were washed three times with TBST (Solarbio, China) at room temperature, with each wash lasting 10 min. Subsequently, the membranes were incubated with horseradish peroxidase-conjugated secondary antibodies (ZSGB-BIO, China) at a 1:1000 dilution for 2 h at room temperature. Immunoreactive signals were detected using an enhanced chemiluminescence (ECL) kit (SparkJade, China). The resulting images were captured and analyzed using ImageJ software (version 1.54).

**Table 2:** Antibody details used in the present study.

Antibody	Source	Dilutions	Company
β-actin	Rabbit	1:100000	Abclonal, Wuhan, China
Reg3γ	Rabbit	1:1000	Affinity Biosciences, United States
pSTAT3	Rabbit	1:1000	Affinity Biosciences, United States
STAT3	Rabbit	1:1000	Affinity Biosciences, United States
ZO1	Rabbit	1:1000	Affinity Biosciences, United States
Occludin	Rabbit	1:1000	Affinity Biosciences, United States
MyD88	Rabbit	1:1000	Affinity Biosciences, United States
HIPPAP	Rabbit	1:1000	Affinity Biosciences, United States

### 2.9 16S rDNA amplicon sequencing

In this study, total DNA was extracted from each sample using the OMEGA Soil DNA Kit (M5635-02) from Omega Bio-Tek, United States. The quality of the extracted DNA was assessed using a NanoDrop spectrophotometer (Thermo Fisher Scientific, USA) and confirmed through agarose gel electrophoresis. Polymerase chain reaction (PCR) amplification targeted the V3–V4 region of the 16S rRNA gene, with universal primers 338F (5′-ACTCCTACGGGAGGCAGCA-3′) and 806R (5′-GGACTACHVGGGTWTCTAAT-3′). The extracted DNA served as the template for the PCR process.

The PCR reaction mixture consisted of 5 μL of 5 × buffer, 0.25 μL of Fast Pfu DNA Polymerase (5 U/μL), 2 μL of deoxynucleotide triphosphates (dNTPs, 2.5 mM), 1 μL of each primer (Forward and Reverse, 10 μM), 1 μL of DNA template, and 14.75 μL of double-distilled water (ddH₂O), resulting in a total volume of 25 μL. The thermal cycling protocol included an initial denaturation step at 98°C for 5 min, followed by 25 cycles of denaturation at 98°C for 30 s, annealing at 53°C for 30 s, and extension at 72°C for 45 s. A final extension step at 72°C for 5 min was then performed.

Following amplification, the PCR products were purified, quantified, and pooled in equimolar concentrations. The pooled products underwent 2 × 250 bp paired-end sequencing using the Illumina NovaSeq platform, performed by Shanghai Huada Gene Biological Technology Co., Ltd. (Shanghai, China).

Bioinformatic analysis of the microbial datasets was carried out using QIIME2 version 2019.4. High-quality Amplicon Sequence Variant (ASV) tables were generated through rigorous quality control, denoising, and clustering processes. Subsequent data analysis was performed using the Gene Cloud platform (accessible at https://www.genescloud.cn).

### 2.10 Statistical analysis

Statistical analyses were conducted using SPSS software, version 22.0. The Shapiro–Wilk test was used to assess the normality of data distributions. Data conforming to a normal distribution were expressed as mean ± standard deviation. For comparisons between two groups, independent sample *t*-tests were used. One-way ANOVA was applied for comparisons among multiple groups, followed by Tukey’s *post hoc* test for pairwise comparisons. For data not meeting the assumptions of normality, the Kruskal–Wallis test was employed. A *p*-value less than 0.05 was considered statistically significant. The sample size was consistent with similar published studies and deemed adequate to detect meaningful differences within the study’s scope (Yin et al., 2015).

## 3 Results

### 3.1 LGG ameliorates high-fat diet-induced MAFLD and related biochemical indices in mice

**Figure 1:**
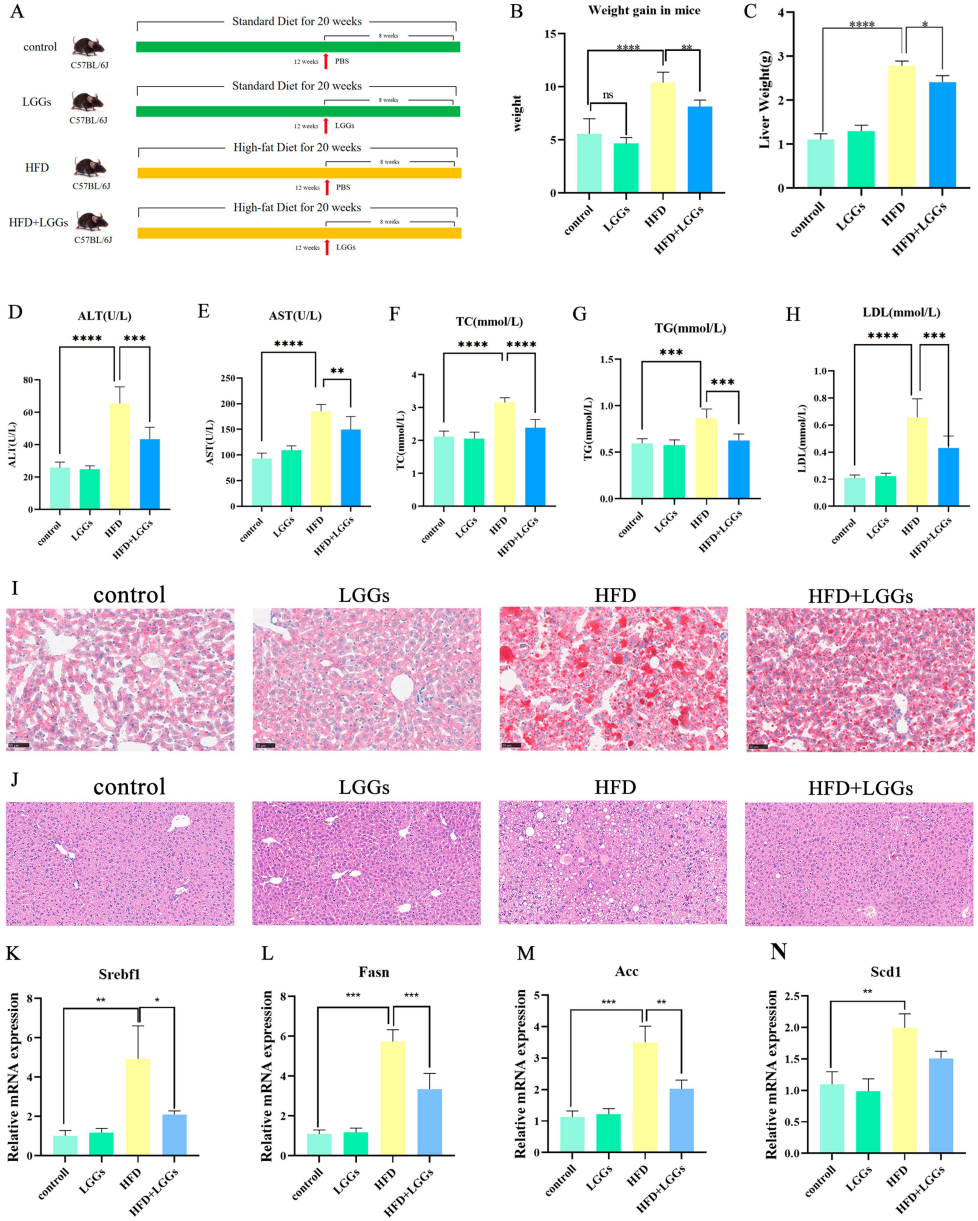
Effects of LGG supernatant on HFD mice. **(A)** Experimental design and treatment procedures. **(B)** Body weight changes in the murine models. **(C)** Liver weights of the mice. (**D–H)** Biochemical parameters in mouse serum, including ALT, AST, TC, TG, and LDL-C. **(I)** Representative images of Oil Red O-stained liver tissues. **(J)** Representative images of H&E-stained liver tissues.**(K–N)** Changes in hepatic lipid metabolism-related gene expression in murine models. LGGs (Lactobacillus rhamnosus GG supernatant); HFD (high-fat diet); ALT (alanine aminotransferase); AST (aspartate aminotransferase); TC (total cholesterol); TG (triglycerides); LDL-C (low-density lipoprotein cholesterol). TG (triglycerides); LDL-C (low-density lipoprotein cholesterol). Data are presented as mean ± standard deviation (SD). *p < 0.05, **p < 0.01, ***p < 0.001.

Following the administration of LGG gavage to MAFLD mice, changes in body weight growth were quantified. A significant reduction in body weight growth was observed in the MAFLD mice treated with LGG compared to untreated MAFLD mice (Figures 1A–C). This finding confirmed the effect of LGG in reducing body weight in mice that had been given a high-fat diet.

Histopathological analysis of Oil Red O-stained liver sections revealed substantial fatty infiltration in the livers of MAFLD mice. In contrast, MAFLD mice treated with LGGs exhibited a significant improvement in hepatic steatosis (Figure 1I). Biochemical analyses, including liver function and blood lipid profiles, further supported these findings, demonstrating that LGG mitigated hepatic injury and significantly reduced lipid levels in MAFLD mice (Figures 1D–H). Hematoxylin and eosin (HE) staining revealed characteristic MAFLD pathology in HFD-fed mice, including macrovesicular steatosis (evident as large, optically clear cytoplasmic vacuoles displacing nuclei) and hepatocellular ballooning (Figure 1J). In contrast,HFD + LGG-treated mice exhibited: Significant attenuation of steatosis: reduced macrovesicular vacuole density and preserved hepatic architecture: Minimal ballooning degeneration and maintained lobular integrity. To assess the effects of HFD and LGG treatment on hepatic lipid metabolism, we quantified the expression of key lipogenic genes: *Sterol Regulatory Element-Binding Transcription Factor 1* (*Srebf1*), the master regulator of hepatic lipid homeostasis implicated in MAFLD pathogenesis; *Fatty Acid Synthase* (*Fasn*), upregulated in metabolic syndrome and MAFLD; *Acetyl-CoA carboxylase* (*Acc*), a crucial enzyme for fatty acid synthesis linked to hepatic insulin resistance; and *Stearoyl-CoA desaturase 1* (*Scd1*), the rate-limiting enzyme for monounsaturated fatty acid production whose inhibition improves hepatic metabolism. qPCR analysis demonstrated a significant upregulation of *Srebf1*, *Fasn*, *Acc*, and *Scd1* mRNA in the livers of HFD-fed mice versus chow-fed controls, confirming the stimulation of hepatic lipogenesis by HFD (Figures 1K–N). Crucially, LGG treatment significantly downregulated the hepatic mRNA expression of all four genes, supporting the therapeutic effect of LGG in mitigating MAFLD-associated lipogenesis.

### 3.2 LGG treatment restores gut microbiota homeostasis in MAFLD mice

**Figure 2:**
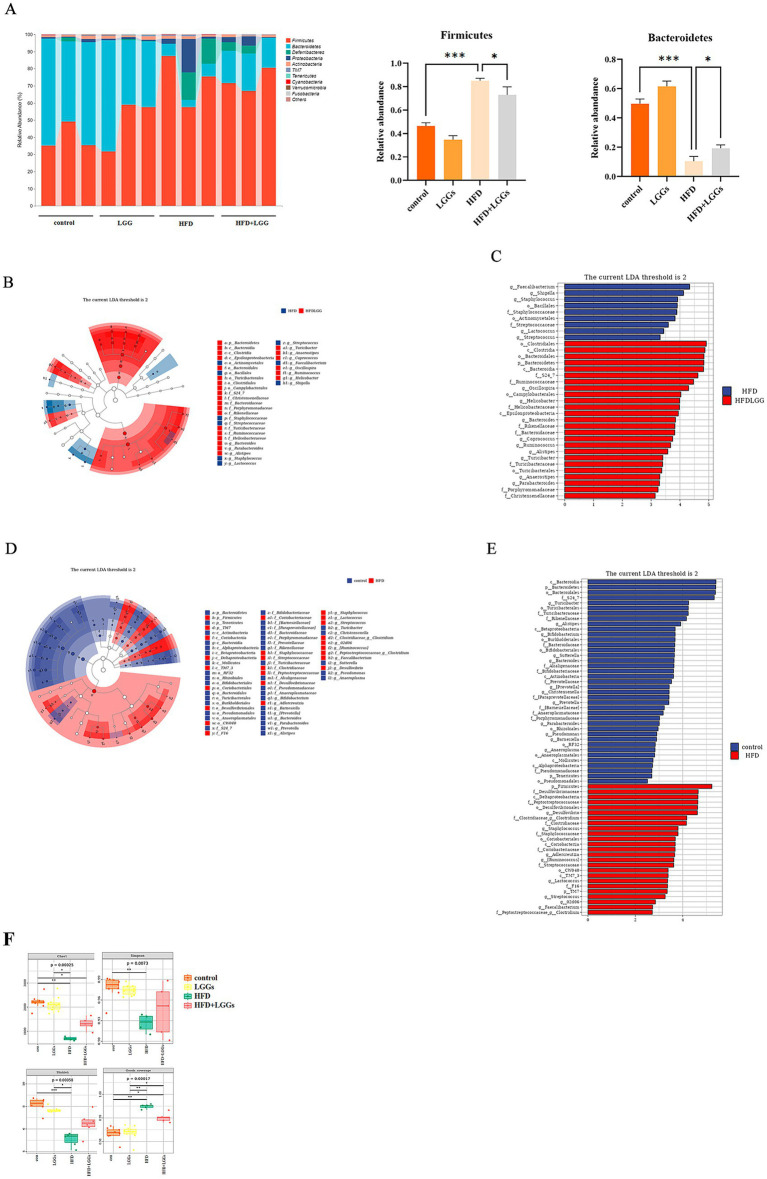
Modulation of gut microbiota by LGGs in mice. **(A)** Fecal microbiota composition across four experimental groups. **(B,C)** LEfSe analysis comparing microbial flora between HFD mice and HFD mice treated with LGGs. **(D,E)** LEfSe analysis comparing microbial flora between standard diet mice and HFD mice. **(F)** Effects of High-Fat Diet and LGGs on Alpha Diversity Indices (Chao1, Shannon, Simpson, Good’s Coverage) of the Mice Gut Microbiota. LGGs (*Lactobacillus rhamnosus* GG supernatant); HFD (high-fat diet).

Microbial sequencing analysis was conducted on fecal samples collected from the experimental mice. The results revealed that *Firmicutes*, *Bacteroidetes*, and *Deferribacteres* were the predominant bacterial phyla in the intestinal tract of the mice. In mice that had been fed an HFD, the abundance of *Bacteroidetes* was significantly reduced, while the abundance of *Firmicutes* was significantly increased compared to those fed a standard diet. Administration of LGG partially restored the disrupted bacterial balance (Figure 2A).

LEfSe analysis further identified that *Firmicutes*, *Desulfovibrionaceae*, and other taxa were more abundant in the HFD group compared to the standard diet group (Figures 2B,C). Conversely, taxa such as *Bacteroidales* and *Oscillospira* were more prevalent in the HFD group treated with LGG. This finding indicates that LGG improved the intestinal environment by modulating the microbiota composition (Figures 2D,E).

Alpha diversity analysis of the gut microbiota revealed significantly decreased indices (including Shannon, Simpson, Chao1, and Good’s coverage) in the HFD group compared to the standard diet group. These findings suggest that high-fat diet reduces alpha diversity in the murine gut microbiome. Notably, LGGs treatment resulted in partial restoration of these alpha diversity indices (Figure 2F).

### 3.3 LGG reduces intestinal barrier dysfunction and endotoxemia in MAFLD

**Figure 3:**
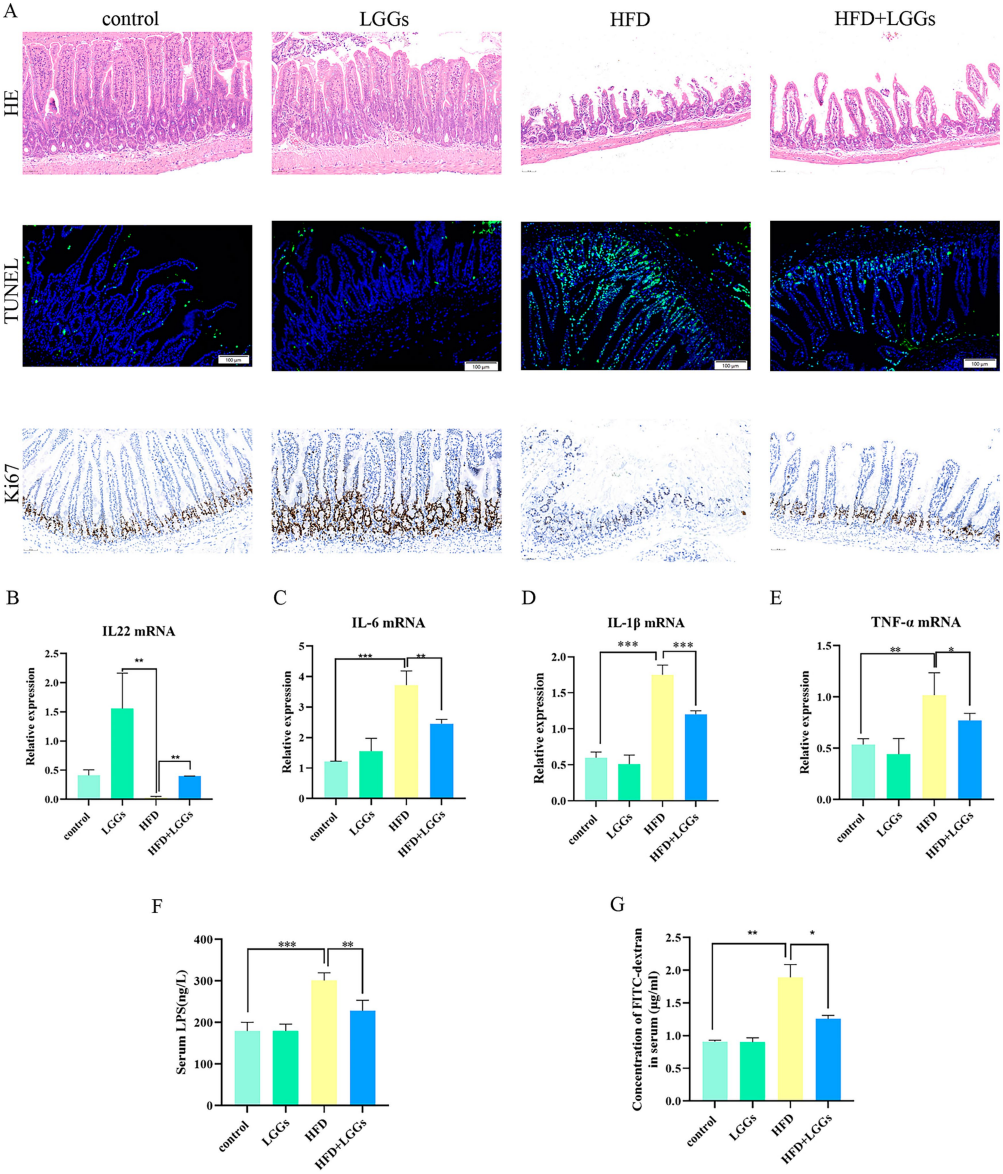
**(A)** Representative images of HE staining, TUNEL immunofluorescence staining, and Ki67 staining of ileal tissues across experimental groups. **(B–E)** Effects of HFD and LGG supernatant on inflammatory cytokine levels (IL-22, IL-6, IL-1β, and TNF-*α*) in ileal tissues. **(F)** Impact of HFD and LGG supernatant on serum LPS levels in mice. **(G)** Effects of HFD and LGG supernatant on intestinal epithelial permeability. HFD (high-fat diet); LGGs (*Lactobacillus rhamnosus* GG supernatant). Data are presented as mean ± standard deviation (SD). *p < 0.05, **p < 0.01, ***p < 0.001.

In HFD-induced obese mice, HE staining of the ileum revealed a disorganized ileocecal villus structure in the HFD group compared to obese mice treated with LGG. Additionally, Ki-67 immunostaining and TUNEL fluorescence assays demonstrated the pro-proliferative and anti-apoptotic effects of LGGs (Figure 3A).

To further assess the impact of LGG on alleviating HFD-induced intestinal inflammation, a significant reduction in pro-inflammatory cytokines, including IL-1β, IL-6, and TNF-α, was observed in ileal tissues of HFD mice following LGG treatment. Conversely, IL-22 levels, which were significantly reduced in HFD mice, were significantly upregulated after LGG administration, either alone or in combination with the HFD (Figures 3B–E).

Further investigation into endotoxemia revealed that serum levels of LPS (Figure 3F) and FITC-dextran (Figure 3G) were significantly reduced in HFD mice following LGG gavage. These findings indicate that LGG improved intestinal integrity in HFD mice.

Collectively, the results confirm that LGG exhibits anti-inflammatory, pro-proliferative, and anti-apoptotic properties, thereby contributing to the preservation of intestinal mucosal barrier in HFD mice.

### 3.4 LGG promotes intestinal Reg IIIγ expression in MAFLD

**Figure 4:**
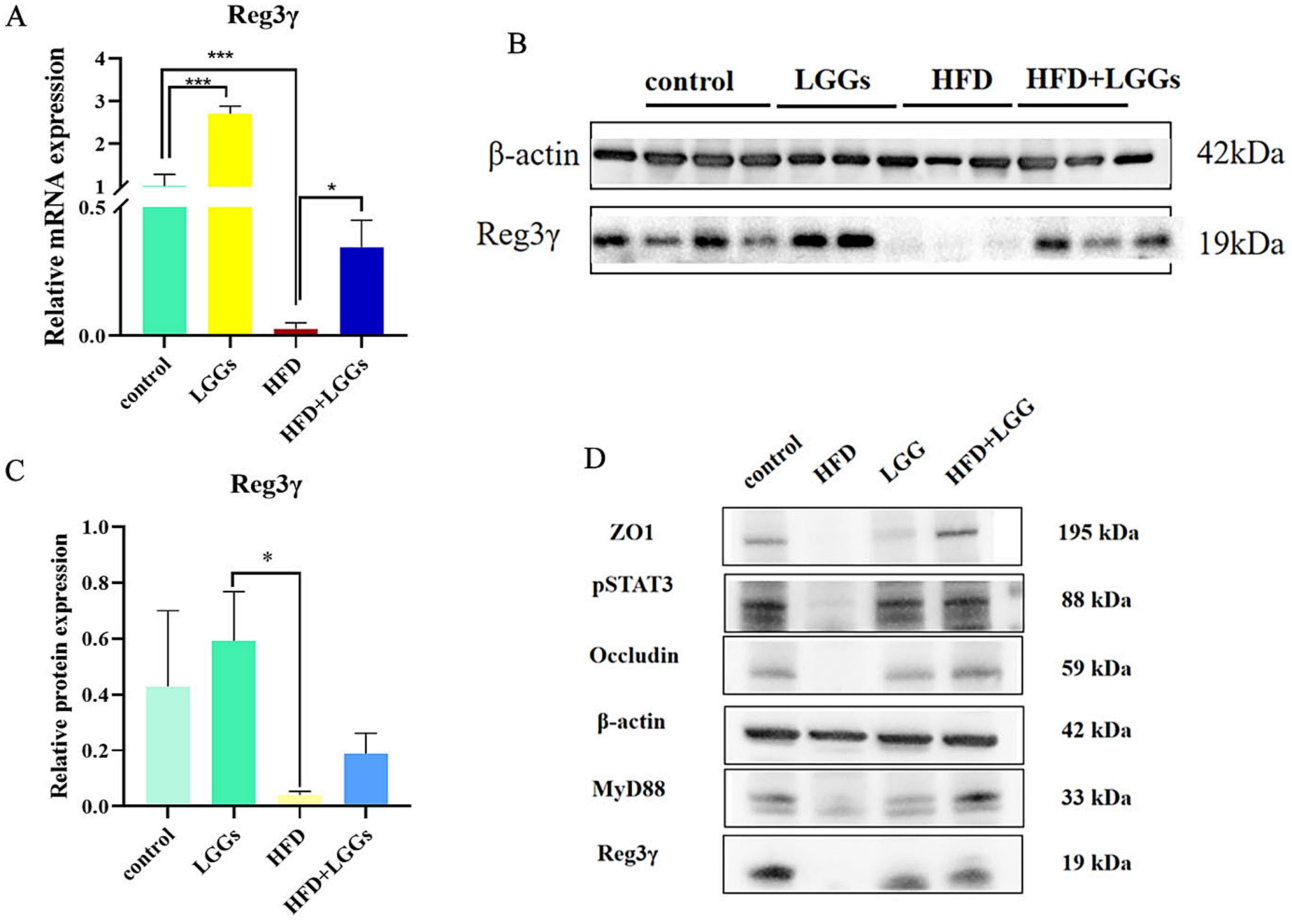
Effect of LGG supernatant on Reg3γ expression in the ileum of HFD mice. **(A)** Relative expression levels of Reg3γ mRNA in ileal tissues across experimental groups. **(B,C)** Relative expression levels of Reg3γ protein in ileal tissues across experimental groups. **(D)** Western blot analysis of Reg3γ, pSTAT3, MyD88, ZO-1, and occludin protein expression in ileal tissues across experimental groups. LGG (*Lactobacillus rhamnosus* GG); HFD (high-fat diet). Data are presented as mean ± SD. *p < 0.05, **p < 0.01, ***p < 0.001.

Further studies into the changes in Reg IIIγ expression in the ileum of HFD mice with and without LGG supplementation revealed a significant reduction in Reg IIIγ expression at both mRNA and protein levels in HFD mice. However, Reg IIIγ expression was restored following LGG gavage in HFD mice (Figures 4A–C). Similarly, the expression levels of intestinal barrier-associated proteins ZO-1 and occludin displayed patterns consistent with Reg IIIγ changes (Figure 4D). These findings, combined with the previous pathological results, indicate a correlation between Reg IIIγ expression, intestinal barrier integrity, and intestinal inflammatory injury.

As indicated by prior studies, the production of Reg IIIγ is closely linked to the pSTAT3/MyD88 signaling pathway (Yin et al., 2015; Xu et al., 2019). Accordingly, pSTAT3 and MyD88 protein expression levels were assessed in the ileal tissues of mice. The findings confirm that Reg3γ expression in the ileum is associated with the pSTAT3/MyD88 pathway.

### 3.5 LGG promotes Reg IIIγ expression through TLR2/MYD88/pSTAT3 pathway in Caco-2 and IEC-6 cells

**Figure 5:**
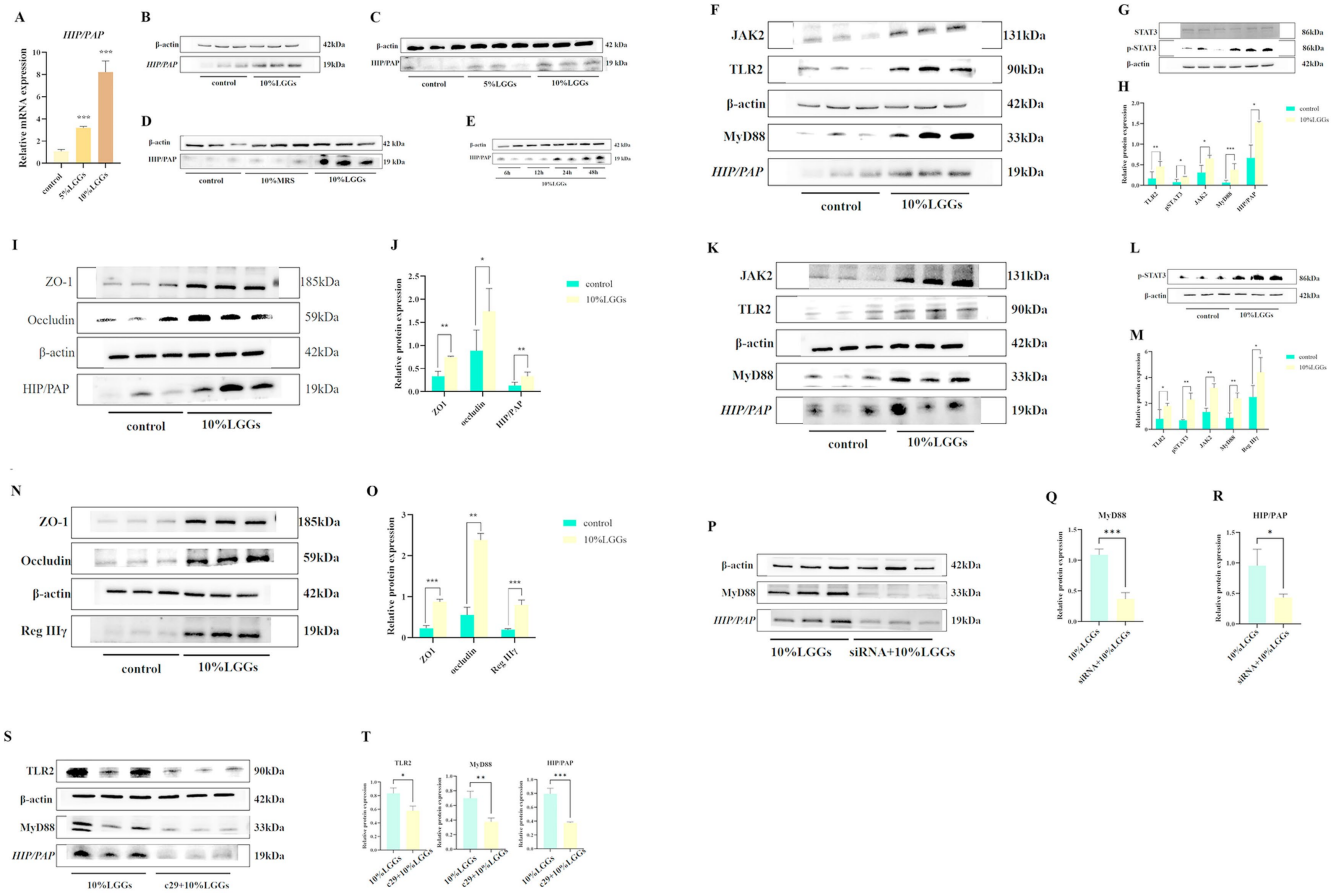
Effects of LGG-conditioned medium on HIP/PAP and related signaling pathways. **(A)** Effect of 24-h stimulation with varying concentrations of LGGs on HIP/PAP mRNA expression in Caco-2 cells. **(B)** Effect of LGGs on HIP/PAP production by Caco-2 cells. **(C)** Effect of 24-h stimulation with 5 and 10% LGGs on HIP/PAP protein expression in Caco-2 cells. **(D)** Effect of 24-h stimulation with 10% MRS or 10% LGGs on HIP/PAP protein expression in Caco-2 cells. **(E)** Effect of 10% LGGs on HIP/PAP protein secretion by Caco-2 cells at different time points. **(F,H)** Effect of 24-h treatment with 10% LGGs on TLR2/MyD88/HIP/PAP pathway in Caco-2 cells. **(G,H)** Effect of 1-h treatment with 10% LGGs on pSTAT3 levels in Caco-2 cells. **(I,J)** Effect of 24-h treatment with 10% LGGs on intestinal barrier-associated protein expression in Caco-2 cells. **(K,M)** Effect of 24-h treatment with 10% LGGs on TLR2/MyD88/HIP/PAP pathway in IEC-6 cells. **(L,M)** Effect of 1-h treatment with 10% LGGs on pSTAT3 levels in IEC-6 cells. **(N,O)** Effect of 24-h treatment with 10% LGGs on intestinal barrier-associated protein expression in IEC-6 cells. **(P–R)** Effect of 10% LGGs on HIP/PAP protein expression in MyD88-knockdown Caco-2 cells. **(S,T)** Effect of 10% LGGs on HIP/PAP protein expression in TLR2-inhibited Caco-2 cells. LGG (*Lactobacillus rhamnosus* GG); HIP/PAP (Hepatocarcinoma-intestine-pancreas/pancreatitis-associated protein). Data are presented as mean ± SD. *p < 0.05, **p < 0.01, ***p < 0.001.

The human homolog of mouse Reg IIIγ, known as HIP/PAP, is expressed in human cells and shares 62–67% sequence similarity with mouse Reg IIIγ (Mukherjee et al., 2014; Chen et al., 2019). HIP/PAP is widely recognized as the human equivalent of mouse Reg IIIγ. Caco-2 cells were stimulated with LGGs at different concentration gradients for 24 h, followed by detection of changes in the mRNA and protein levels of HIP/PAP in Caco-2 cells. First, Caco-2 cells were stimulated with LGGs at 5 and 10% concentrations for 24 h, and the expression levels of HIP/PAP mRNA and protein were detected using qPCR and Western blot. It was confirmed that 10% concentration of LGGs significantly increased the secretion of HIP/PAP by intestinal epithelial cells (Figures 5A–D). Subsequently, Caco-2 cells were stimulated with 10% LGGs for 6 h, 12 h, 24 h, and 48 h. The results showed that 10% LGGs stimulation for 24 h could significantly increase HIP/PAP levels (Figure 5E). Enhanced HIP/PAP expression was associated with TLR2/MyD88 activation and STAT3 phosphorylation (Figures 5F–J), a finding corroborated in the IEC-6 cell line (Figures 5K–O). To further validate pathway dependency, we knocked down MyD88 expression in Caco-2 cells using siRNA. This ablation abolished the ability of LGGs to promote HIP/PAP production (Figures 5P–R). Complementarily, pretreatment of Caco-2 cells with the TLR2 inhibitor C29 (100 μM, 1 h) significantly attenuated the subsequent HIP/PAP-inducing effect of LGGs (10%, 24 h) (Figures 5S–T). Collectively, these findings demonstrate that LGGs promotes HIP/PAP production primarily through the TLR2/MyD88/STAT3 signaling pathway.

## 4 Discussion

**Figure 6:**
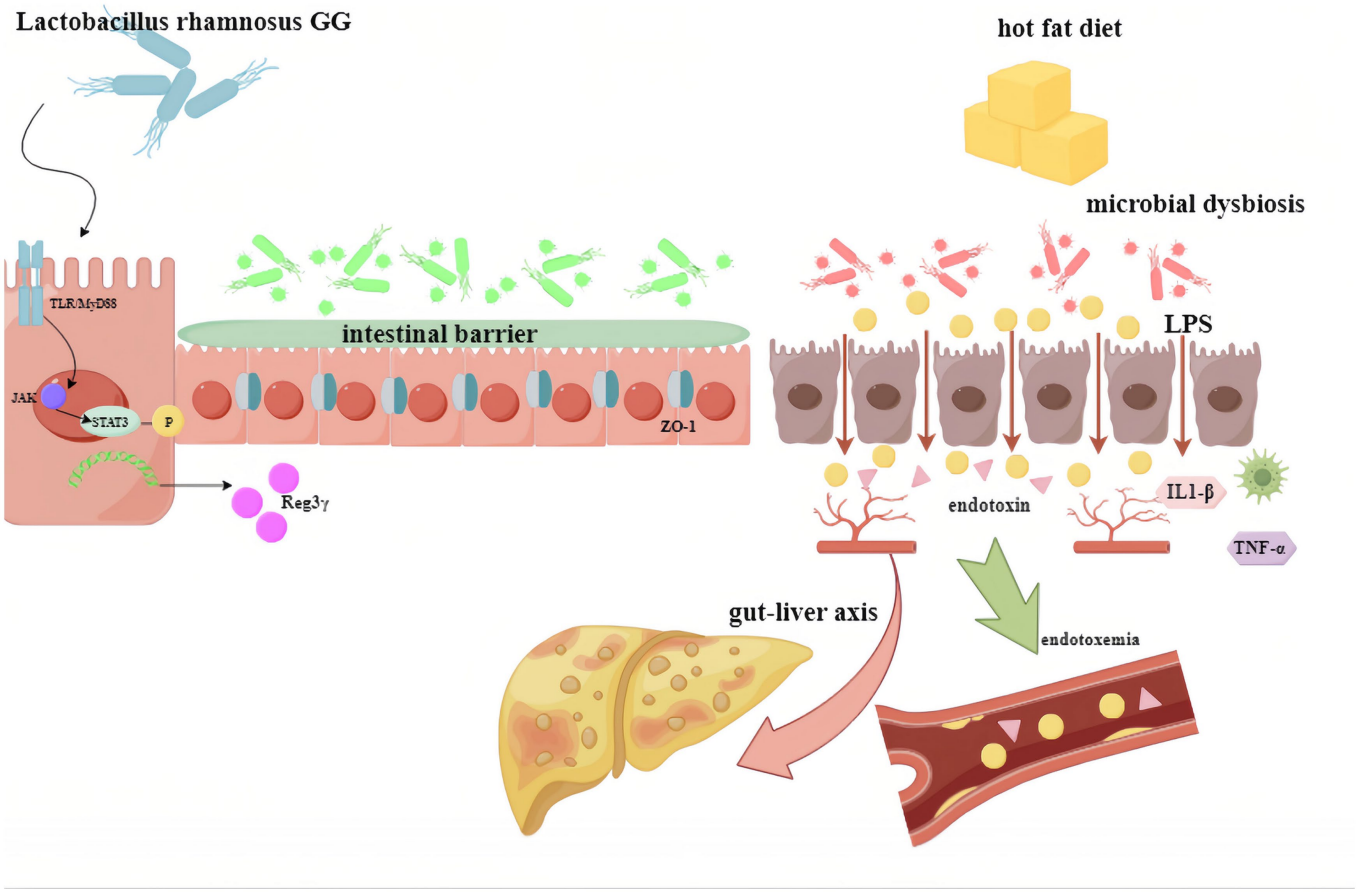
Proposed mechanism depicting how LGG supernatant promotes Reg3γ expression to protect against fatty liver through the pSTAT3/MyD88 signaling pathway. LGG (*Lactobacillus rhamnosus* GG).

The findings of this study demonstrate that supplementation with LGG supernatant alleviated MAFLD induced by a HFD in mice. This effect was achieved through the restoration of intestinal barrier function and mitigation of increased intestinal permeability (“leaky gut”), a process associated with the antimicrobial peptide Reg IIIγ. LGG supernatant supplementation significantly increased Reg IIIγ production, thereby improving intestinal barrier integrity in MAFLD. This promotion of Reg IIIγ expression by LGG supernatant was mediated via the MyD88/pSTAT3 signaling pathway (Figure 6).

HFD disrupts the intestinal epithelial barrier, resulting in changes in gut microbiota composition, increased intestinal permeability, and translocation of LPS produced by *Proteobacteria* into the intestinal lumen (Malesza et al., 2021). The penetration of LPS activates colonic immune responses and disrupts tryptophan-indole pathway metabolism, thereby reducing activation of AHR, which are critical regulators of intestinal barrier homeostasis (Ji et al., 2019; Huang et al., 2022). Consequently, expression levels of the tight junction proteins ZO-1 and occludin were downregulated in the ileal tissues of HFD-fed mice, as observed in this study.

The Reg gene family, initially identified in studies on pancreatitis and pancreatic proliferation, comprises a class of antimicrobial peptides. These genes are expressed across diverse organs and tissues—including the heart, brain, skin, pancreas, liver, nervous system, and intestines—with notably elevated expression within the digestive system (22727489, 23401489). The Reg III protein family includes several subtypes. Murine models feature four distinct members: Reg IIIα, Reg IIIβ, Reg IIIγ, and Reg IIIδ. In humans, however, only two Reg III family members have been identified to date: Reg IIIA (also known as HIP/PAP) and Reg IIIG (10526060). HIP/PAP shares ~ 67% homology with murine Reg IIIγ and has been used as a functional surrogate in prior studies (24015602). To address inconsistencies in nomenclature for homologous Reg proteins across species—and the resulting challenges in cross-referencing or potential interchangeable use of human-rodent Reg homologs—studies on intestinal antimicrobial peptides frequently regard human HIP/PAP and murine Reg IIIγ as functional equivalents (26866937, 36240758).

Previous research indicates that small molecules produced by specific lactic acid bacteria enhance intestinal Reg IIIγ production, likely through MyD88 activation and STAT3 phosphorylation (Frazier et al., 2022). STAT3 phosphorylation promotes the regeneration of intestinal stem cells and facilitates the production of antimicrobial peptides, thereby contributing to the repair of the intestinal mucosa (Sekikawa et al., 2010; Zheng et al., 2008). These findings align with the reduced Reg IIIγ expression observed in the ileal tissues of HFD mice, underscoring the necessity of pSTAT3 activation for Reg IIIγ secretion in the ileum. MyD88, a key adaptor protein in innate immune signaling, plays a pivotal role in intestinal inflammation. Furthermore, pSTAT3 is recruited to the TLR/MyD88 signaling pathway, linking immune signaling to metabolic regulation (Balic et al., 2020).

In this study, the promotion of HIP/PAP in intestinal epithelial cells by LGG supernatant was significantly diminished following siRNA-mediated knockdown of MyD88 expression. Reg IIIγ, expressed throughout the small intestine, plays a key role in maintaining the spatial barrier between gut microbiota and host tissues as part of the innate immune response (Vaishnava et al., 2011; Mukherjee et al., 2014). Bacterial colonization is a major stimulus for Reg IIIγ production under physiological conditions, with its expression influenced by various metabolic conditions (Cash et al., 2006; Yan et al., 2011; Tremblay et al., 2017). This study found that administration of LGGs was associated with increased expression of Reg IIIγ in ileal tissue. Specifically, compared with untreated high-fat diet mice, high-fat diet mice treated with LGG supernatant exhibited significantly elevated levels of Reg IIIγ in their ileal tissue. This finding is consistent with previous reports indicating that Lactobacillus can induce Reg IIIγ expression, suggesting that certain active components of LGG (present in the supernatant) play a crucial role in regulating Reg IIIγ expression (Shin et al., 2022; Shin et al., 2023).

In this study, HFD mice supplemented with LGG supernatant exhibited increased Reg IIIγ expression in their ileum compared to HFD mice without supplementation. The upregulation of Reg IIIγ contributed to the repair of intestinal mucosal injury, a key factor in the pathogenesis of MAFLD, leading to significant improvements in intestinal integrity and a notable amelioration of MAFLD-related damage.

The findings of this study indicate that the expression of Reg IIIγ in the ileum is closely associated with the repair of intestinal mucosa facilitated by LGG. Previous studies reported similar results, demonstrating that metabolic disorders induced by factors such as high-fat diets or alcohol led to the downregulation of intestinal Reg IIIγ expression, resulting in impaired regulation of lipid and glucose metabolism. Conversely, various types of bariatric surgery enhance Reg IIIγ expression (Shin et al., 2022).

Reg IIIγ plays a protective role in intestinal injuries, such as colitis, by exerting anti-inflammatory effects, reducing oxidative stress, and preventing apoptosis (Mukherjee et al., 2014; Zheng et al., 2008). Furthermore, Reg IIIγ limits bacterial translocation in the intestine, protecting against ethanol-induced progression from steatohepatopathy to steatohepatitis (Wang et al., 2016; Miki et al., 2012). These findings support the growing consensus that Reg IIIγ positively influences the intestinal environment by promoting bactericidal activity and survival-related signaling.

This study confirmed that LGG supernatant provides a protective effect against intestinal damage caused by high lipid intake, primarily through the upregulation of Reg IIIγ. However, the precise mechanisms by which LGG activates MyD88 to induce Reg IIIγ expression remain unclear. To address this, the active components of LGG supernatant need to be isolated and purified to identify the specific agents responsible for its protective effects. Moreover, the cross-referencing and potential interchangeability of human and rodent Reg homologs present additional challenges in interpreting findings and comparing them with other studies. Further research is required to address these limitations and provide deeper insights into the mechanisms underlying these effects.

This study reveals a significant inverse correlation between ileal Reg IIIγ expression levels and the severity of MAFLD as well as endotoxemia, a finding with important translational implications. It not only suggests that Reg IIIγ expression levels (or those of its human functional homolog, HIP/PAP) could serve as a potential biomarker for assessing intestinal barrier dysfunction and disease progression in human MAFLD patients, but more importantly, demonstrates that administration of the supernatant from LGG effectively ameliorates MAFLD pathology. This provides direct experimental evidence supporting the development of novel cell-free therapeutic strategies based on the probiotic secretome. Compared to live-bacterium therapies, such approaches circumvent the potential risks associated with live bacterium transplantation, offering greater clinical safety. Examples include the future exploration of oral probiotic-derived biologics or engineered delivery systems. The core mechanism identified in this study—ameliorating gut-derived endotoxemia and its driven hepatic inflammation and metabolic dysregulation by promoting Reg IIIγ (HIP/PAP) expression to restore the intestinal barrier—provides a new theoretical foundation and a potential intervention target for treating MAFLD by targeting the “gut-liver axis.” Building on this, future research should focus on in-depth characterization of the key bioactive components within the LGGs. This necessitates the systematic analysis of the LGGs using metabolomics and proteomics technologies to identify the core effector molecules regulating Reg IIIγ (HIP/PAP) expression. Of particular interest is determining whether known LGG-secreted proteins (such as p40 and p75) mediate the observed biological effects; this requires functional validation using gene-knockout bacterial strains or purified proteins/specific antibodies. Furthermore, optimizing delivery strategies for active components (e.g., nano-encapsulation) to enhance oral bioavailability and intestinal targeting, and validating the protective role of HIP/PAP and its downstream signaling pathways (e.g., STAT3, TLR) in more human-relevant models (such as humanized mice or organoids), will be critical steps in translating these findings toward clinical applications.

The results indicate a strong connection between the liver and intestine through multiple pathways, highlighting that improving intestinal barrier function in MAFLD can mitigate disease progression. This can be achieved by preventing excessive microbial products, such as LPS, and microbial metabolites from entering the liver and systemic circulation. Reg IIIγ, an antimicrobial peptide, contributes to intestinal barrier repair via the MyD88/pSTAT3 pathway, thereby alleviating MAFLD.

Despite the promising findings, several limitations of this study should be acknowledged. Firstly, our findings are primarily derived from a single dietary-induced MAFLD mouse model. While this model recapitulates key features of human MAFLD, the absence of validation in genetic knockout models limits our ability to definitively establish the specificity and absolute necessity of the identified Reg IIIγ pathway in mediating the protective effects of LGG supernatant. Secondly, in our *in vitro* experiments utilizing human HIP/PAP as a functional homolog for murine Reg IIIγ, we did not account for the inherent pH difference between LGGs and standard bacterial growth medium (MRS). This omission means we cannot entirely rule out the possibility that the observed effects on HIP/PAP expression or barrier function in cell lines were partially influenced by pH changes rather than solely by bioactive components within LGGs. Thirdly, the reliance on murine Reg IIIγ in the *in vivo* setting and human HIP/PAP *in vitro* introduces a significant translational consideration. Given the known species-specific differences in Reg III family members (notably the absence of a direct Reg IIIγ ortholog in humans), extrapolating the precise mechanistic role of Reg IIIγ observed in mice directly to the human context requires caution, although HIP/PAP is recognized as the closest functional analog. Fourthly, a major design limitation is the absence of a control group receiving sterile MRS medium alone. Since the complete LGG supernatant was administered orally, the control group should have received the vehicle in which the bacterial metabolites are found (i.e., MRS medium), to distinguish effects attributable specifically to LGG-derived factors from those potentially caused by residual components of the culture medium. Finally, prior to assessing HIP/PAP expression and barrier function in cell lines treated with LGGs, we did not perform comprehensive cytotoxicity assays (e.g., CCK-8). While no overt toxicity was observed morphologically, the lack of quantitative cytotoxicity data means we cannot conclusively exclude the possibility that changes in gene expression or barrier metrics were indirectly influenced by subtle alterations in cell viability or proliferation induced by LGGs components or pH.

## 5 Conclusion

In conclusion, this study demonstrated the protective effects of LGG on fatty liver. The intervention repaired the intestinal mucosal barrier and reduced intestinal inflammation by upregulating Reg IIIγ expression, a process closely linked to STAT3 phosphorylation and involving MyD88. These findings indicate that this strategy offers potential benefits for the treatment of HFD-induced liver disease and associated intestinal injury by promoting Reg IIIγ production in the intestine. Further investigations are needed to elucidate the precise role of Reg IIIγ in colitis.

## Data Availability

The original contributions presented in the study are publicly available. This data can be found here: NCBI SRA, accession number: PRJNA1303743.

## Glossary

LGG: *Lactobacillus rhamnosus* GG
MAFLD: Metabolic associated fatty liver disease
LGGs: *Lactobacillus rhamnosus* GG supernatants
PBS: Phosphate-buffered saline
HFD: High-fat diet
HIP/PAP: Hepatocarcinoma-intestine-pancreas/pancreatitis-associated protein
MRS: DeMan, Rogosa, and Sharpe
ALT: Alanine aminotransferase
AST: Aspartate aminotransferase
TC: Total cholesterol
TG: Triglycerides
LDL-C: Low-density lipoprotein cholesterol
LPS: Lipopolysaccharides
FITC: Fluorescein isothiocyanate
HE: Hematoxylin and eosin
TUNEL: TdT-mediated dUTP Nick-End Labeling
PVDF: Polyvinylidene difluoride
TBST: Tris-Buffered Saline with Tween-20
IL-1β: Interleukin-1β
IL-6: Interleukin-6
IL-22: Interleukin-22
TNF-α: Tumor necrosis factor-α
